# Functional Connectivity Hubs and Networks in the Awake Marmoset Brain

**DOI:** 10.3389/fnint.2016.00009

**Published:** 2016-03-04

**Authors:** Annabelle M. Belcher, Cecil Chern-Chyi Yen, Lucia Notardonato, Thomas J. Ross, Nora D. Volkow, Yihong Yang, Elliot A. Stein, Afonso C. Silva, Dardo Tomasi

**Affiliations:** ^1^Department of Psychiatry, University of Maryland School of MedicineBaltimore, MD, USA; ^2^National Institute on Drug Abuse, National Institutes of HealthBaltimore, MD, USA; ^3^National Institute of Neurological Disorders and Stroke, National Institutes of HealthBethesda, MD, USA; ^4^National Institute on Drug Abuse, National Institutes of HealthRockville, MD, USA; ^5^National Institute on Alcohol Abuse and Alcoholism, National Institutes of HealthBethesda, MD, USA

**Keywords:** resting-state fMRI, functional connectivity, hubs, resting-state networks

## Abstract

In combination with advances in analytical methods, resting-state fMRI is allowing unprecedented access to a better understanding of the network organization of the brain. Increasing evidence suggests that this architecture may incorporate highly functionally connected nodes, or “hubs”, and we have recently proposed local functional connectivity density (*l*FCD) mapping to identify highly-connected nodes in the human brain. Here, we imaged awake nonhuman primates to test whether, like the human brain, the marmoset brain contains FC hubs. Ten adult common marmosets (*Callithrix jacchus*) were acclimated to mild, comfortable restraint using individualized helmets. Following restraint training, resting BOLD data were acquired during eight consecutive 10 min scans for each subject. *l*FCD revealed prominent cortical and subcortical hubs of connectivity across the marmoset brain; specifically, in primary and secondary visual cortices (V1/V2), higher-order visual association areas (A19M/V6[DM]), posterior parietal and posterior cingulate areas (PGM and A23b/A31), thalamus, dorsal and ventral striatal areas (caudate, putamen, lateral septal nucleus, and anterior cingulate cortex (A24a). *l*FCD hubs were highly connected to widespread areas of the brain, and further revealed significant network-network interactions. These data provide a baseline platform for future investigations in a nonhuman primate model of the brain’s network topology.

## Introduction

The last decade has witnessed a precipitous increase in the number of fMRI reports exploring patterns of correlated signals that occur spontaneously in the human brain. The most reproducible of these patterns are seen in regions that form functionally related circuits consistent with diffusion-weighted fMRI-based measures of structural connectivity and with nonhuman primate neuroanatomical tract-tracing work, and are referred to as resting state networks (RSNs; Damoiseaux and Greicius, 2009; Greicius et al., 2009; Kelly et al., 2010; Margulies et al., 2010; Choi et al., 2012). Observed even when subjects are not engaged in task-directed behaviors, these “resting-state” fMRI (rsfMRI) networks are in constant flux, and are thought to individually reflect synchronous activity (with neurophysiological underpinnings) of brain regions participating in concert to produce integrative brain functions (Smucny et al., 2014).

Several of these networks have been identified in humans and animals, and are named by the functional localization and/or cognitive domain construed to be relevant (for example, executive, salience, visual and sensorimotor networks). Additionally, alterations in several of these networks have been reported for a variety of conditions including Alzheimer’s disease, depression, and addiction (Lustig et al., 2003; Anand et al., 2005; Sutherland et al., 2012), suggesting that this approach may serve as a tool for the identification of aberrant, disease-related circuitry that could potentially serve as a biomarker for clinical disorders (Buckner et al., 2013; Castellanos et al., 2013; Fedota and Stein, 2015). This notion is not uncontested however; it is well-known that vagaries in laboratory data collection and analysis methods, as well as task and state-dependent factors can figure heavily in the results and interpretation of resting-state data (Cole et al., 2010; McAvoy et al., 2012; Power et al., 2012; Shirer et al., 2012). In a thoughtful and critical review of the utility of RSfMRI, Buckner et al. (2013) argue that intrinsic functional connectivity uniquely provides information about relations between networks at a *whole-brain* level: a feature which, at minimum, merits its use as a tool for generating testable hypotheses that can only be explored with external methods of probing brain function. For ethical reasons, these methods preclude studies with humans, demanding more research with preclinical models. And high-order cognitive processes, such as those that are under putative study with the use of RSfMRI, are best modeled in animals whose behaviors and neuroanatomical complexity are most proximate to humans: nonhuman primates.

Several elegant studies have reported RSfMRI studies in nonhuman primates (Vincent et al., 2007; Hutchison et al., 2011, 2012), and a few have even gone the step further to combine fMRI techniques with invasive approaches to better understand resting-state (O’Reilly et al., 2013; Wang et al., 2013). With only a handful of exceptions, however (Moeller et al., 2009; Mantini et al., 2011, 2013), most RSfMRI studies in primates have been carried out under the confounding effects of anesthesia. We have recently reported successful application of an awake imaging protocol for acquisition of fMRI data in marmoset monkeys (Silva et al., 2011). Due to their small size and ease with which they can be handled and trained, marmosets provide an excellent neuroscience model for explorations of RSfMRI that are able to be followed up with more invasive probes of brain function. Using spatially independent component analysis methods to investigate RSNs in marmosets, we found that the brain of this small New World nonhuman primate possesses functionally relevant network patterns that are generally similar to those found in Old World non-human primates and humans (Belcher et al., 2013). The networks we reported included several sensory and “low-order” networks, as well as several “high-order” cognitive and association networks, including those analogous to the human default mode and salience networks.

Increasing evidence indicates that like many complex systems (Barabási, 2009), the network architecture of the human brain may incorporate highly functionally connected nodes, or “hubs”; an idea that is consistent with small-world and scale-free network models of brain function (Bullmore and Sporns, 2012). Using a novel algorithm, we previously provided evidence to support the notion that the normal human brain follows this organizational principle of hubs of connectivity (Tomasi and Volkow, 2010, 2011). The application of novel data-driven analysis methods of rsfMRI to preclinical models—especially those in nonhuman primates that are likely to more accurately represent the functional organization of the human brain—provides an important step in the direction of providing superior preclinical platforms for the testing of notions of rsfMRI. A long-standing choice model for biomedical and drug development research, the common marmoset is currently enjoying a surge in popularity as an ideal model for systems neuroscience. The clear advantages afforded vis-à-vis its compact size, strong social and familial bonding behaviors, innate intelligence and efficient reproductivity was enough to convince the Japanese government to fund a major 10-year brain mapping project with the marmoset as the center stage model (Brain/MINDS; Cyranoski, 2014). The fact that the marmoset is the only nonhuman primate shown to produce viable expression of a transgene in its offspring (Sasaki et al., 2009) cements its superiority as a biomedical research model.

The aim of the current study was to employ data-driven functional connectivity density mapping (FCDM) to identify whether, like the human brain, the conscious marmoset brain possesses FC hubs, brain regions with high local functional connectivity density (*l*FCD). Additionally, we used standard seed-voxel correlation analyses using the hubs as defined seeds to study the FC to these hubs with the whole-brain. The results provide weight to the rationale of using a model that characterizes more aptly the scale-free organization of the mammalian brain.

## Materials and Methods

### Subjects

All experiments were approved by the Animal Care and Use Committee of the National Institute of Neurological Disorders and Stroke (NINDS). A total of ten adult male common marmosets between the ages of 2–4 years old and weighing 350–425 g were used in this study. Marmosets were housed in same-sex pairs in cages with a 12 h light/dark cycle and fed twice daily on a diet of Zupreem canned marmoset food, Purina 5040 food biscuits, unfiltered water, BioServ P.R.A.N.G. oral rehydrator, and various fruits and vegetables.

### Awake Training

Training and awake imaging restraint procedures and materials have been described elsewhere (Silva et al., 2011). Briefly, within a 3-week training period, animals were progressively exposed to behavioral acclimation in three phases: (1) acclimation to atraumatic restraint with a jacket and plastic cover plate and placement (sphinx position) in a mock scanner environment; (2) restraint as in phase 1, but with the addition of MRI sounds played during the training; and (3) restraint as in phase 2, but with individualized plastic helmets to minimize head motion. A behavioral rating scale (Schultz-Darken et al., 2004) was used on each training day to assess the individual animals’ tolerance to the acclimatization procedure; all monkeys successfully completed the training with behavioral rating scores of ≤4 and proceeded to the imaging phase of the study.

### BOLD Data Acquisition

MRI scans were obtained on a single day session in a 7T/30 cm USR horizontal Bruker scanner using a custom-built birdcage volume coil and a custom-built 4 cm double circular loop surface coil placed on top of the helmet. Local magnetic field inhomogeneity estimation and shimming were performed based on field map measurements for magnetic field optimization. Functional imaging data was acquired using a gradient-echo EPI sequence with the following parameters: TE/TR = 25/1500 ms, FOV 45 mm, matrix 80 × 80, 400 timepoints, slice thickness 2 mm and 15 coronal slices. Eight 10 min-long single-shot resting state scans were collected for each monkey. In addition, a T_2_-weighted structural scan was acquired for each animal with the same geometric parameters as the EPI, but with a 160 × 160 matrix (four averages; TE/TR = 72/6000 ms).

### Preprocessing

Functional data was preprocessed in AFNI (NIMH/NIH; Cox, 1996) and included skull-stripping, slice timing correction, and registration to a base EPI volume. Each individual animal’s anatomical (T_2_-weighted) scan was registered to the EPI time series, and spatially normalized to a common space using a high-resolution T_2_ scan of a single marmoset brain (150 μm isotropic voxels; TE/TR = 40/500 ms), and resliced using 0.5 mm in plane resolution and 2 mm slice thickness.

### Local Functional Connectivity Density (*l*FCD)

The interactive data language (IDL, ITT Visual Information Solutions, Boulder, CO, USA) was used for subsequent FCDM. A multilinear regression approach was used to minimize motion related fluctuations (Tomasi and Volkow, 2010) and standard 0.01–0.08 Hz band-pass temporal filtering was applied to remove magnetic field drifts and minimize high frequency physiological noise components in the EPI time series. The *l*FCD at every voxel in the brain was computed as the number of elements in the local FC cluster, using a “growing” algorithm (Tomasi and Volkow, 2010). Pearson correlation was used to assess the strength of the FC, *R_ij_*, between voxels *i* and *j* in the brain, and a correlation threshold of *R_ij_* > 0.2 was selected to ensure that correlations between time-varying signal fluctuations were significant at *p* < 0.0001 (two-tailed). A voxel (*x_j_*) was added to the list of voxels functionally connected with *x*_0_ only if it was adjacent to a voxel that was linked to *x*_0_ by a continuous path of functionally connected voxels (faces touching) and *R*_0*j*_ > 0.2. This calculation was repeated for all brain voxels that were adjacent to those that belonged to the list of voxels functionally connected to *x*_0_ in an iterative manner until no new voxels could be added to the list. Eight different *l*FCD maps, corresponding to the 8 EPI timeseries, were averaged for each animal. Within-subjects analysis of variance (ANOVA) was used to assess the statistical significance of the *l*FCD in the brain. Statistical significance was set by a voxel-level P_FWE_ < 0.05, corrected for multiple comparisons with the random field theory and a family-wise error correction, using SPM8 (Wellcome Trust Centre for Neuroimaging, London, UK). Anatomical regions were labeled according to Paxinos et al. (2011).

### Hub (Seed)-Voxel Correlations

We next mapped networks functionally connected to the above identified functional *l*FCD hubs (regions with high *l*FCD). The 16 identified *l*FCD hub (see “Results” Section) allowed precise definition of four cortical and four subcortical seeds comprising two *l*FCD-hubs each (left and right) were used as seed regions (18 voxels per seed; 9 voxels per hub; total seed volume 9 μl) for subsequent seed-voxel correlation analyses (Table 1). The spatial coordinates of the seeds were kept fixed across all animals, and placement was confirmed by visual inspection on an individual subject basis. The Fisher’s *r*-to-*z* transform was used to normalize the FC of the voxel with the seed. The eight different seed-voxel maps were averaged independently for each animal and seed. The averaged maps from all seed regions and animals were analyzed using a within-subjects ANOVA in SPM8. The statistical significance of the FC patterns was based on a cluster-level P_FWE_ < 0.05, family wise error corrected for multiple comparisons using the random field theory. A cluster-forming threshold of *p* < 0.005 and a minimum cluster size of 100 voxels were used for this purpose.

**Table 1:** **Spatial location, strength (*k*) and statistical significance (*T*-score) of the *l*FCD hubs in the marmoset brain**.

Region		Coordinates [mm]			*l*FCD	
	Seed#	*x*	*y*	*z*	*k*	*T*

V1	1	0.75	−18.00	3.05	396	21.1
V2		0.60	−18.00	4.10	373	19.6
A19M	2	0.90	−16.65	4.55	441	20.7
V6(DM)		0.60	−13.80	5.30	477	18.1
PGM	3	0.30	−12.60	6.35	467	15.0
A23b/A31		0.15	−9.15	7.40	432	14.7
THA	4	−3.00	−6.00	0.65	482	8.8
THA		3.15	−6.00	1.10	532	9.0
PU	5	−5.10	−1.80	2.15	596	11.4
PU		6.15	−1.80	2.15	580	14.7
CD	6	−3.15	0.00	3.50	577	12.5
CD		5.40	0.00	3.20	554	13.9
A24a	7	−1.65	0.00	6.35	590	13.5
A24a		1.80	0.00	6.35	587	13.8
LSI	8	−1.20	0.15	2.00	501	12.6
LSI		1.35	0.15	2.30	585	13.8

*The anterior commissure (AC) was used as the center of the Cartesian coordinate system. The connection of AC and posterior commissure (PC) in the middle of the brain forms the y-axis. The x-axis runs from the left to the right hemisphere through AC. The z-axis runs from the inferior part of the brain to the superior part through the AC (n.b.: coordinates listed in this manuscript can be readily converted to Paxinos’ coordinate system by subtracting 9.5 mm, the approximate distance between the center of the AC and ear canal)*.

### Network-Network Correlations

Average signal time courses were computed within each FC pattern for each EPI scan and animal. The Pearson correlation and the Fisher’s *r*-to-*z* transform were used to compute FC matrices assessing the linear association between average time courses for each EPI scan and animal. The FC matrices corresponding to the 8 EPI scans were averaged independently for each animal. Within-subjects ANOVA was used to assess the statistical significance of the correlation matrix across subjects using a Bonferroni correction for multiple comparisons of *p* < 0.05/7/4 = 0.001786.

## Results

### Functional Hubs

**Figure 1:**
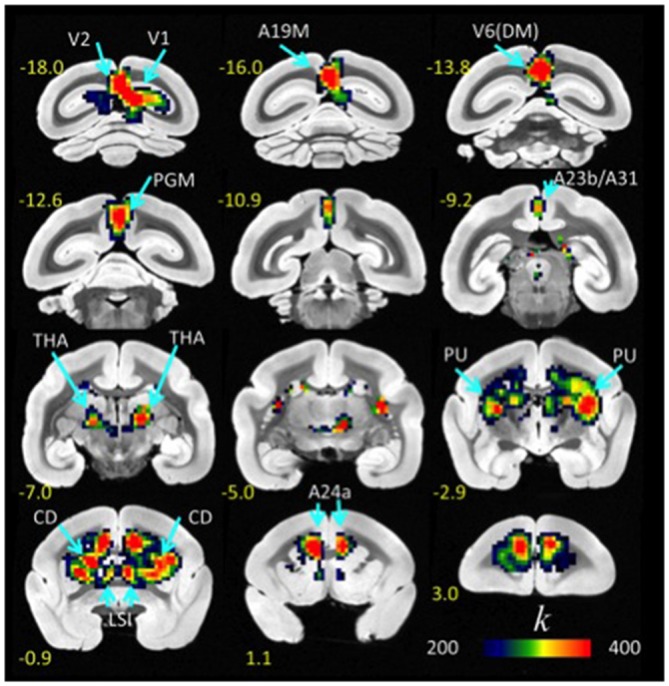
**Average *l*FCD distribution.** Spatial distribution of the *l*FCD superimposed on coronal T2 MRI views of the marmoset brain. The most prominent *l*FCD hubs were located in V1 and V2, the primary and secondary visual areas; A19M, the medial part of cortical area 19; V6(DM), the dorsomedial part (DM) of visual area 6 (V6); PGM, the medial part of parietal area; PG, posterior cingulate areas A23b and A31; THA, thalamus; PU, putamen and CD, caudate; LSI, the intermediate part of the lateral septal nucleus; and cingulate area A24a. These maps reflect the average number of functional connections per voxel (*k*) across 10 awake marmosets. Yellow numbers indicate the distance in mm from the anterior commissure (AC).

FCDM revealed prominent *l*FCD hubs across the cortex, including visual cortex (primary visual cortex, V1; visual area 2, V2; dorsomedial visual area 6, V6(DM); medial part of area 19, A19M), medial parietal cortex (PGM), posterior (A23b/A31) and anterior (A24a) cingulate cortices, as well as subcortical regions (caudate, CD; putamen, PU; lateral septal nucleus, intermediate part, LSI and the thalamus [anterior pulvinar nucleus and posterior, thalamic nuclear group; THA]). In these regions (Figure 1, Table 1), the *l*FCD was higher than the whole brain average *l*FCD.

**Figure 2:**
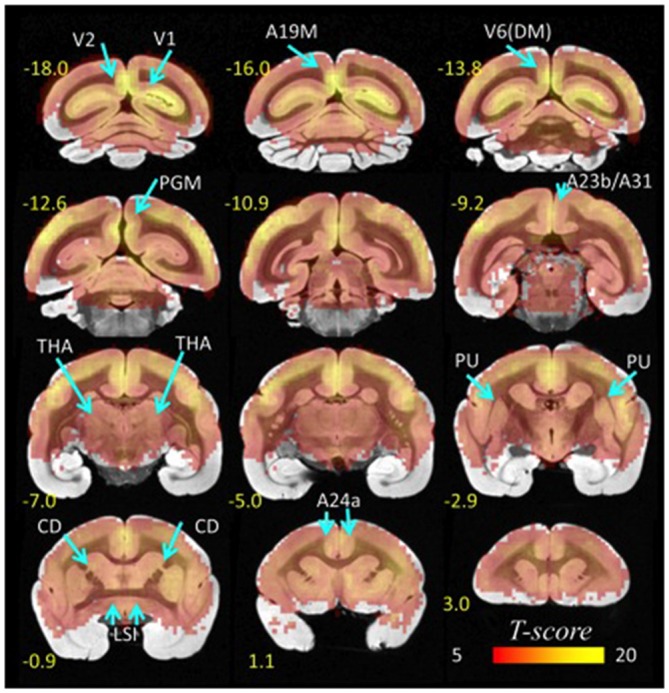
**Statistical significance of *l*FCD superimposed on coronal T2 MRI views of the marmoset brain.** The most prominent *l*FCD hubs were located in V1 and V2, the primary and secondary visual areas; A19M, the medial part of cortical area 19; V6(DM), the DM, dorsomedial part of visual area 6 (V6); PGM, the medial part of parietal area; PG, posterior cingulate areas A23b and A31, THA, thalamus; PU, putamen and CD, caudate; LSI, the intermediate part of the lateral septal nucleus; and cingulate area A24a. Statistical model: within-subjects ANOVA; statistical threshold: P_FWE_ < 0.05.

The probability of the *l*FCD, P(k), decreased exponentially with *k*, the strength of the *l*FCD, such that there were few highly connected hubs and numerous weakly connected nodes, a pattern that was highly significant across the eight repetitions and ten animals (*p* < 0.001, *T*-score > 3). Furthermore, the *t*-values of the *l*FCD were larger for cortical regions than for subcortical regions, as within-subject variability in the strength of *l*FCD was higher for the subcortical hubs than for the cortical hubs (Figure 2). This finding reflects the predominance of highly dynamic local connectivity in these cortical brain regions in the marmoset (standard deviation-to-mean ratio; i.e., relative variability- of the *l*FCD = 49 ± 21% for CD and 27 ± 10% for V1; *p* < 0.006, paired *t*-test, df = 9).

### Resting-State Hub Networks (Seed-Voxel Correlations)

**Figure 3:**
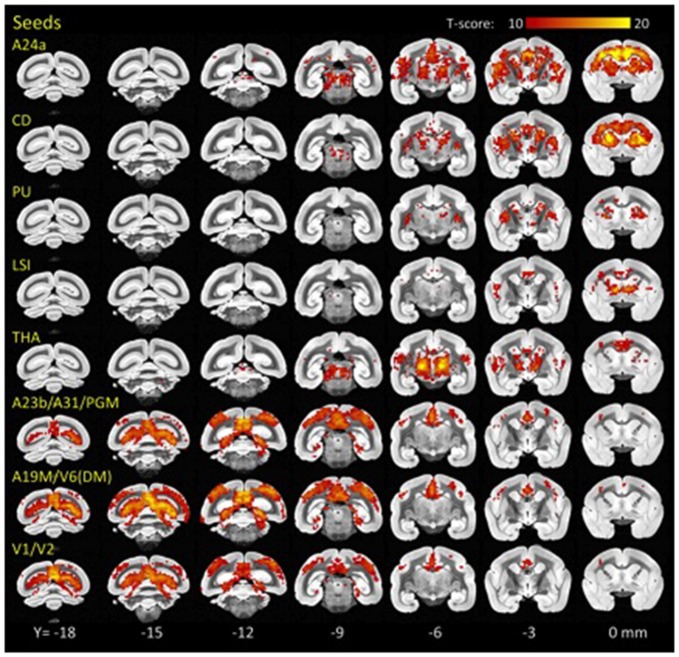
**Hub-specific patterns of FC.** Statistical significance of seed-voxel correlation patterns computed for each *l*FCD hub (yellow labels), superimposed on coronal MRI views of the marmoset brain. Numbers indicate the distance in mm to AC. Statistical model: within-subjects ANOVA. Statistical threshold: P_FWE_ < 0.05.

Using standard seed-voxel correlation analyses we identified eight bilateral networks functionally connected to the *l*FCD hubs (all regions identified in Figure 3). When these networks were thresholded at P_FWE_ < 0.05, their union covered 90% of the brain volume. The FC patterns revealed different functional networks (Figure 3). Specifically, anterior brain seed regions (A24a, LSI, CD, PU and THA) demonstrated positive correlation patterns that overlapped in subcortical regions (basal ganglia and thalamus). Conversely, seeds located in posterior regions of the brain demonstrated correlation patterns that overlapped in cortical regions (visual areas, posterior cingulate and retrosplenial cortex).

Specifically, the network functionally connected to bilateral *l*FCD hubs in A24a included bilateral anterior cingulate, premotor, motor and somatosensory cortices, granular and dysgranular insula, thalamus and the striatum. *l*FCD hubs in CD demonstrated bilateral functional connectivity with frontal cortical regions including the anterior cingulate, premotor, motor and somatosensory cortices, CD, claustrum, PU and thalamus. The network functionally connected to the bilateral *l*FCD hubs in PU included bilateral PU, CD, claustrum and thalamus, and that connected to LSI included bilateral septum, medial and lateral accumbens shells, ventral pallidum, substantia innominata (basal part), lateral preoptic area, median and medial preoptic nuclei, septohypothalamic nucleus, dorsal nucleus of the endopiriform claustrum and the insular cortex. The *l*FCD hubs in THA showed functional connectivity with bilateral thalamus, anterior cingulate, insula and primary auditory cortex. The networks functionally connected to the posterior *l*FCD hubs (A23b/A31/PGM; A19M/V6(DM); V1/V2) included anterior and posterior cingulate and medial parietal cortices, V6(DM), PGM, anterior, medial, ventral and lateral intraparietal areas, parietal areas PE, PG and PFG and motor, somatosensory, retrosplenial and visual cortices.

### Network-Network Interactions

**Figure 4:**
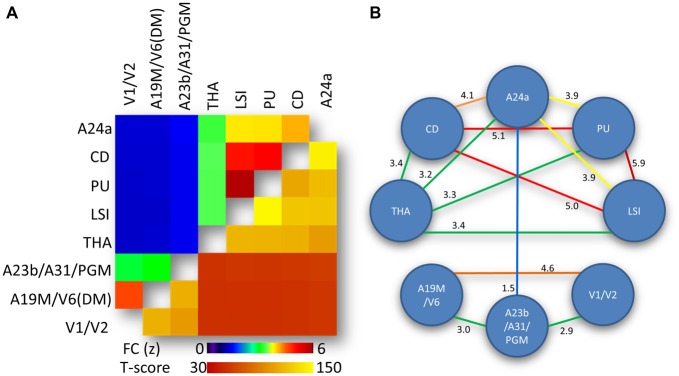
**Network-network interactions. (A)** Average correlation matrix (superior-left triangle of the symmetric correlation matrix) reflecting the FC between average time courses from the eight hub-specific networks in Figure 2 and the corresponding statistical significance (inferior-right triangle of the symmetric statistical matrix). Statistical model: within-subjects ANOVA. **(B)** Diagram highlighting predominant (yellow-red) and moderated (green-blue) interactions between hub-specific networks. Weak network-network interactions (FC < 1.5) are not shown. Numbers quantify the strength of the FC between the networks.

The average correlation matrix revealed significant interactions between the eight bilateral hub-specific networks (*p* < 0.00001; Figure 4A, lower right triangular matrix). Specifically, two modules were identified based on the strength of the correlation between ROI time courses: an anterior module (LSI, PU, CD, THA and A24a) and a posterior module (A23b/A31/PGM, A19M/V6(DM) and V1/V2), which exhibited *FC*_(*z-*score)_ > 3 (Figure 4A, upper left triangular matrix), as depicted by the diagram’s connecting lines (edges) in Figure 4B. For the anterior module the FC was maximal between PU and LSI, and minimal between A24a and THA. For the posterior module the FC was maximal between A19M/V6(DM) and V1/V2, and minimal between A23b/A31/PGM and V1/V2. The edges between A23b/A31/PGM and A24a mediated the interaction between the anterior and posterior modules (Figure 4B).

## Discussion

RsfMRI and its relevance to brain circuitry and disease has revolutionized neuroimaging research. Arguably, one of the greatest strengths of this task-independent approach lies in its translational utility. Here, we show for the first time the distribution of brain FC hubs in awake marmosets. In total, we identified eight hubs of maximal connectivity; specifically: (1) anterior cingulate, A24a; (2) caudate, CD; (3) putamen, PU; (4) lateral septal nucleus, LSI; (5) thalamus,THA; (6) retrosplenial cortex, A23b and A31; and the medial aspect of posterior parietal area PG, PGM; (7) midline visual areas A19M and V6(DM); and (8) primary and secondary visual cortical areas (V1/V2).

### General Patterns of Connectivity

The most prominent *l*FCD hubs in the marmoset brain were located in anterior cingulate (A24a) and subcortical (LSI, CD, PU and THA) regions, findings which contrast with the predominance of *l*FCD hubs in the posterior regions of the human brain (Tomasi and Volkow, 2010, 2011). Other *l*FCD hubs in the marmoset brain were located in occipital [V1, V2, A19M, V6(DM)], parietal and posterior cingulate (PGM, A23b, A31) cortices (Figures 1, 2). Additionally, cortical hubs in marmosets showed high local connectivity, as *l*FCD was stronger for cortical regions than for subcortical regions.

We identified the overall FC patterns using the hub locations as seed regions, in a subsequent connectivity analysis. Our study demonstrates specific pathways from *l*FCD hubs that reveal the overall functional architecture of the marmoset brain, consistent with the important role of *l*FCD hubs in the architecture of the brain. Of translational interest, the marmoset brain showed prominent hubs with wide connectivity patterns that reflect those found in the human brain; specifically, visual networks, a thalamic network, and a network that corresponds to the human default mode network (DMN).

The finding of a strong visual hub of connectivity corresponds well with our previous finding of several robust resting-state visual networks based on Independent Component Analysis (ICA; Belcher et al., 2013). Marmosets are highly visual organisms that display acute tracking and visual recognition behaviors (Mitchell and Leopold, 2015), with the neural real estate devoted to primary visual processing encompassing nearly 20% of this animal’s total cortical volume (Rosa and Tweedale, 2005), so perhaps it is not surprising that we found these areas to be epicenters of activity. Additionally, we found a hub centering on the posterior cingulate cortex, which constitutes the core area of the human DMN (Buckner et al., 2008). The posterior cingulate/precuneus, in particular, has been identified as a particularly highly connected hub in human resting-state data, with strong state-independent connections to all other structures of the DMN (Buckner et al., 2009). A hub similar to this (with local maxima in the restrosplenial cortex) has been identified in rodent resting-state data (Lu et al., 2012), suggesting that this network is a cross-species phenomenon. Consistent with this, we showed that the hub in the posterior cingulate gyrus served as the functional connection between the posterior and the anterior networks. The DMN has been postulated as a system of brain regions that is most active when the brain is in an “idle” mode and is particularly identifiable (and is maximally engaged) in humans (Buckner et al., 2008) and monkeys (Mantini et al., 2011) during between-trial periods of directed task behavior.

Additionally, we found the anterior cingulate cortex to be a hub in the marmoset brain, with high connectivity to a network of structures that, in the human, have been identified as the “salience network.” First described in human resting state data by Seeley et al. (2007) and frequently observed in subsequent reports, this network has been only intermittently observed in anesthetized nonhuman primate data (a “cinguloinsular” component; Hutchison et al., 2011) and in our awake marmoset model (Belcher et al., 2013).

Finally, we found statistically significant subcortical hubs that centered on the caudate, putamen and thalamus, each of which we have previously reported as networks in the marmoset brain. In our previous report, this component very faithfully tracks the anatomical borders of the caudate and putamen, and skips the fiber bundle (internal capsule) that runs between the two structures (Belcher et al., 2013). This anatomical fidelity and very well-understood afferent and efferent circuitry make this network and hub a particularly appealing region of the marmoset brain for study. Interestingly, the caudate hub showed strong connectivity with frontal cortical regions, with significant overlap with regions connected to the anterior cingulate (A24a).

Other groups have reported findings suggesting that, as is the case with the human brain, functional network topography in the Old World nonhuman primate brain follows a modular organization characterized by rich, densely connected hubs of connectivity (Harriger et al., 2012; Scholtens et al., 2014). Constrained by the underlying neural architecture, resting-state functional connectivity has been found to be most stable in regions with reciprocal structural connections (i.e., rich club cores; Shen et al., 2015). These data in New World monkeys provide evidence to suggest that these organizing principles are an evolutionarily-conserved feature of the primate brain.

### Network-Network Interactions

As previously reported in the human brain (Bell and Shine, 2015) we showed a strong convergence between functional resting networks in the marmoset RSNs (Figure 3). The strongest network overlap existed between hubs contained within the striatum (CD, PU, LSI, all with FC ≥ 5.0; Figure 4B). Another strong network-network interaction was found between the two visual hubs, that of V1/V2 and A19/V6, and a moderate level of connectivity was observed between the anterior (A24) and posterior (A23/A31/PGM) aspects of the cingulate cortex.

## Limitations

A limitation of our study is that we did not control or interfere with the subjects’ arousal level. Anecdotally, the subjects’ eyes tended to remain closed during scan sessions, opening intermittently and at pulse sequence onsets. Shifting levels of arousal could perhaps have varying effects on rsfMRI, as patterns of cortical connectivity, although identifiable (Fransson et al., 2007; Vincent et al., 2007), are attenuated during sleep and light anesthesia (Massimini et al., 2007; Greicius et al., 2008). Yet even during slow-wave sleep, marmoset cortical excitability is far superior to that obtained under general anesthesia (Issa and Wang, 2011), underscoring the clear advantage of the awake marmoset platform for measuring patterns of brain connectivity.

In the current report, *l*FCD is restricted to the local functional connectivity cluster and does not assess degree (also called global FCD or gFCD), which is computationally demanding at high spatiotemporal resolution (Tomasi and Volkow, 2010). However, this is not a strong limitation because previous studies have shown that the *l*FCD and degree metrics are proportional to one another, and are both related to energy consumption (Tomasi and Volkow, 2010; Tomasi et al., 2013).

Although the precise methods may vary from one lab to another, approaches to rsfMRI data analysis can be grouped into one of two categories: those that are hypothesis-driven (for example, seed-based analyses, whereby an *a priori* region of interest is defined and correlations with that region’s activity are assessed), and those that are data-driven (for example, ICA, whereby minimally overlapping, orthogonal patterns of activity are identified across the brain). Using these techniques, although a few networks observed in humans have been identified in rodents (Becerra et al., 2011; Upadhyay et al., 2011; Lu et al., 2012), most of the RSNs identified in humans have been faithfully recapitulated in nonhuman primates; particularly for the high-order cognitive resting-state signatures (Vincent et al., 2007; Hutchison et al., 2011; Mantini et al., 2012; Belcher et al., 2013). We can only speculate that this difference reflects the decreased phylogenetic distance between humans and non-human primates; but whatever the reason, nonhuman primate data are likely to translate better to humans.

These data are consistent with results obtained using this same platform of awake imaging (Belcher et al., 2013). In that report, ICA revealed 12 networks obtained from the resting-state data of six conscious marmoset monkeys. The networks found included several visual, somatomotor, striatal, and high-order cognitive networks, including a salience and DMN. Here, we obtained resting-state data from 10 conscious marmosets to ask a slightly more nuanced question: do principles of human brain organization (i.e., scale-free [Barabási and Albert, 1999]) apply to the marmoset brain? Several studies have shown that human brain networks have scale-free and small-world properties (Watts and Strogatz, 1998; Barabási and Albert, 1999). In this study we show that the probability distribution of *l*FCD hubs in the marmoset brain has scale-free properties similar to those found in the human brain (Tomasi and Volkow, 2010). Our findings support the notion that highly-connected areas of the nonhuman primate brain likely represent highly-trafficked regions of neuronal activity, and that these hub regions could provide the infrastructure for circuit communication.

In conclusion, we present a novel approach to the analysis of rsfMRI data in conscious marmoset monkeys. Our data are consistent with a scale-free topology (Barabási and Albert, 1999), and with the distribution of *l*FCD in the human brain (Tomasi and Volkow, 2010), broadly mirroring findings of hubs of high connectivity in the human brain.

## Author Contributions

AMB, CC-CY, LN and ACS performed experiments; AMB, LN, TJR, YY and DT analyzed the data; and AMB, NDV, EAS and DT wrote the manuscript.

## Funding

Intramural Research Programs of the National Institute of Neurological Disorders and Stroke (NINDS), National Institute on Alcohol Abuse and Alcoholism (NIAAA), and the National Institute on Drug Abuse (NIDA), NIH.

## Conflict of Interest Statement

The authors declare that the research was conducted in the absence of any commercial or financial relationships that could be construed as a potential conflict of interest. The handling Editor declared a shared NIH affiliation with the authors CC-CY, LN, TJR, NDV, YY, EAS, ACS, DT and states that the process nevertheless met the standards of a fair and objective review.
